# Supplementary material for: Dual membrane receptor degradation via folate receptor targeting chimera
Source: Nat Commun. 2025 Oct 2;16:8804. doi: 10.1038/s41467-025-63882-5 (PMC12491540; doi:10.1038/s41467-025-63882-5)
Supplement: Supplementary file 1 — Supplementary Information [file 41467_2025_63882_MOESM1_ESM.pdf]

Dual Membrane Receptor Degradation via Folate Receptor Targeting Chimera

Zhen Wang, Zhixin Li, Jenny Högström, Hiroyuki Inuzuka, Rui Jing, Peiqiang Yan, Tao Hou, Yihang Qi,

Daoyuan Huang, Jingchao Wang, Ting Wu, Xiaoying Shi,

Bolin Liu, Taru Muranen, Dingpeng Zhang✉, Wenyi Wei✉

✉ **Corresponding author**

dzhang13@bidmc.harvard.edu (D.Z.);

wwei2@bidmc.harvard.edu (W.W.).

This file includes:

Supplementary Figs. 1 to 9

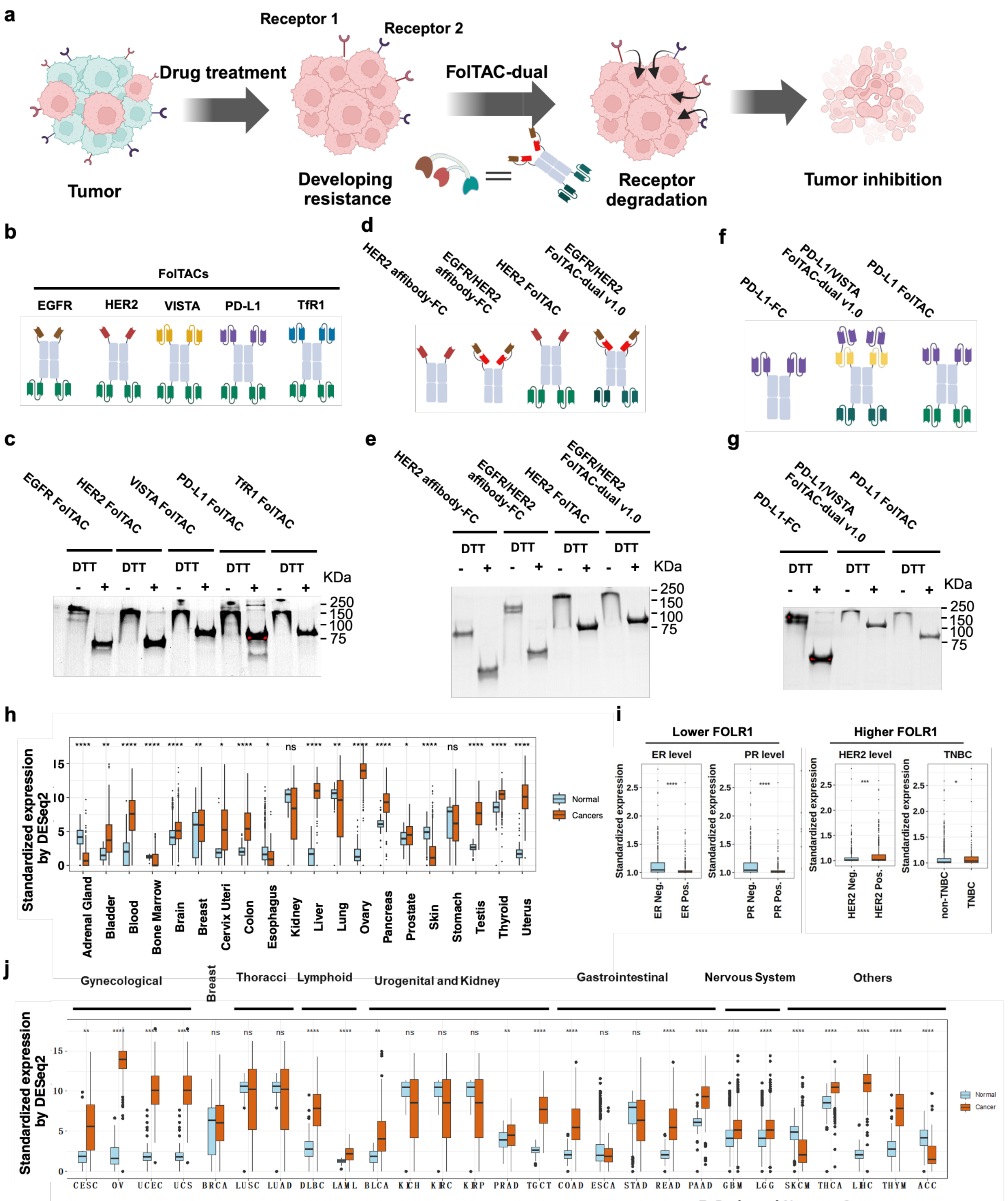

1. Gynecological
•CESC: Cervical and Endocervical Cancers;
•OV: Ovarian Serous Cystadenocarcinoma;
•UCEC: Uterine Corpus Endometrial Carcinoma;
•UCS: Uterine Carcinosarcoma;

2. Breast
•BRCA: Breast Carcinoma;

3. Thoracic (Lung cancer)
•LUSC: Lung Squamous Cell Carcinoma;
•LUAD: Lung Adenocarcinoma;

4. Hematopoietic and Lymphoid
•DLBC: Diffuse Large B-cell Lymphoma;
•LAML: Acute Myeloid Leukemia;

5. Urogenital and Kidney (male specific)
•BLCA: Bladder Urothelial Carcinoma;
•KICH: Kidney Chromophobe;
•KIRC: Kidney Renal Clear Cell Carcinoma;
•KIRP: Kidney Renal Papillary Cell Carcinoma;
•PRAD: Prostate Adenocarcinoma;
•TGCT: Testicular Germ Cell Tumors;

6. Gastrointestinal
•COAD: Colon Adenocarcinoma;
•ESCA: Esophageal Carcinoma;
•STAD: Stomach Adenocarcinoma;
•READ: Rectum Adenocarcinoma;
•PAAD: Pancreatic Adenocarcinoma;

7. Brain and Nervous System
•GBM: Glioblastoma Multiforme;
•LGG: Brain Lower Grade Glioma;

8. Other cancers:
Skin
•SKCM: Skin Cutaneous Melanoma;
Endocrine
•THCA: Thyroid Carcinoma;
Liver
•LIHC: Liver Hepatocellular Carcinoma;
Thoracic
•THYM: Thymoma;
Head and Neck
•ACC: Adenoid Cystic Carcinoma;

Supplementary Fig. 1. SDS-page gel characterization of different FolTAC designs, and transcriptomic profile of FRα expression level comparison between normal and cancer tissues.

a, A schematic illustration of FolTAC-dual for drug resistance.

b, A schematic illustration of the five different FolTACs.

c, Protein gel analysis of FolTACs in (b).

d, Scheme of EGFR/HER2 FolTAC-dual and the control antibody.

e, Protein gel analysis of FolTACs and control antibody in (d).

f, Scheme of PD-L1/VISTA FolTAC-dual and the control antibody.

g, Protein gel analysis of FolTACs and control antibody in (f).

h, Transcriptomic analysis of FRα expression in different normal and cancer tissues.

i, Transcriptomic profile of *FOLR1* expression in Estrogen Receptor (ER), Progesterone Receptor (PR), Human Epidermal Growth Factor Receptor 2 (HER2), and Triple-Negative Breast Cancer (TNBC) Low and High Cancer.

j, Transcriptomic profile of *FOLR1* expression in different cancer subtypes.

Exact P value for Supplementary Fig. 1h, 1i, and 1j are provide in Zenodo under the DOI: https://doi.org/10.5281/zenodo.16421330. Normal cells are indicated in blue, and cancer cells are indicated in orange. Created in BioRender. WANG, Z. (2025) https://BioRender.com/d5n9vj6.

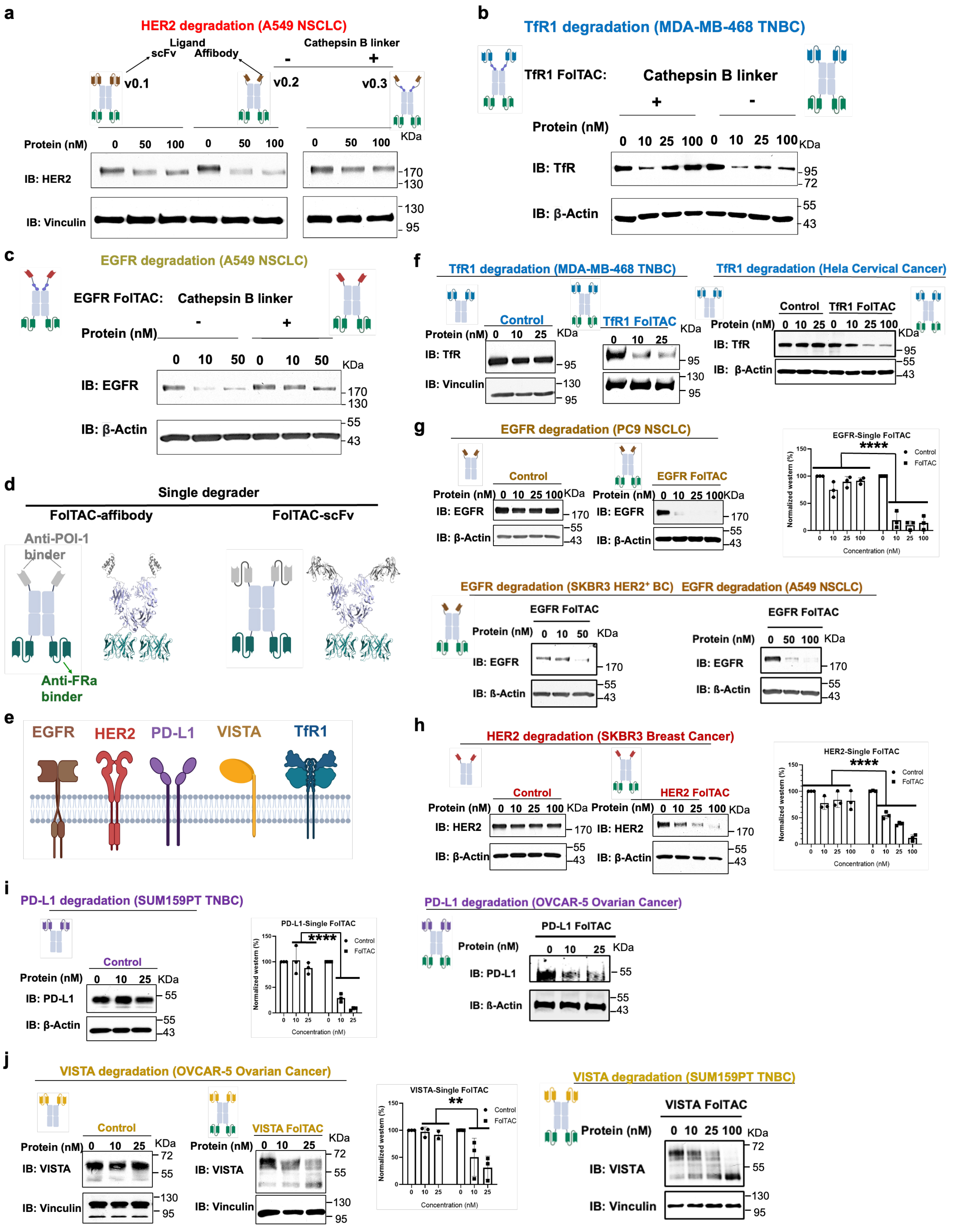

Supplementary Fig. 2. Optimization and subsequent analysis of FolTAC-mediated degradation of EGFR, HER2, PD-L1, or VISTA.

a, Western blotting of HER2 degraded by HER2 Trastuzumab (Traz) FolTAC v.01, HER2 affibody v0.2, HER2 affibody with Cathepsin B linker (CatB) v0.3 in A549 cells. Relative protein levels were labeled with numbers below the indicated bands.

b, Western blotting of TfR1 degraded by TfR1 FolTAC or TfR1 FolTAC-CatB in MDA-MB-468 cells.

c, Western blotting of EGFR degraded by EGFR FolTAC or EGFR FolTAC-CatB in A549 cells.

d, Schematics, and structures of FolTAC.

e, Schematics of various cell surface targets degraded by their respective FolTACs.

f-j, Western blots and quantifications showing the degradation of (f) TfR1 on MDA-MB-468 TNBC and HeLa cervical cancer cells; (g) EGFR on PC9, A549 NSCLC, and SKBR3 breast cancer; (h) HER2 on SKBR3 breast cancer; (i) PD-L1 on SUM159PT TNBC and OVCAR-5 ovarian cancer; (j) VISTA on OVCAR-5 ovarian cancer and SUM159PT TNBC. Data are shown as mean ± SD. n = 3 independent biological replicates. Statistical significance was determined using two-sided unpaired Student's t-tests. Exact *p*-values are indicated above the bars. Differences were considered statistically significant at $p < 0.05$. **, ****: $p < 0.01$, $p < 0.0001$. The PD-L1 degradation data used for statistical analysis in Supplementary Fig. 2i are included in the main Fig. 3g.

Created in BioRender. WANG, Z. (2025) https://BioRender.com/d5n9vj6.

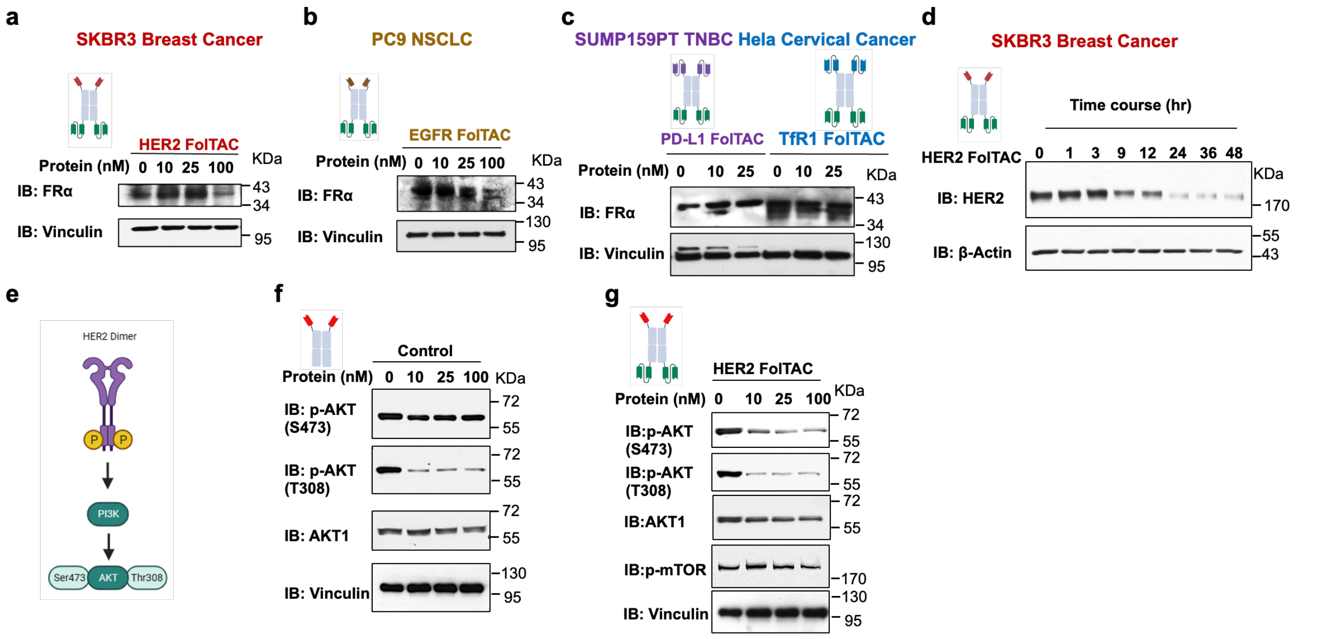

Supplementary Fig. 3. Mechanism study of single FolTAC treatment in cancer cells using various indicated degraders.

a, Western blot analysis of FRα levels in SKBR3 cells treated with HER2 FolTAC.

b, Western blot analysis of FRα levels in PC9 cells treated with EGFR FolTAC.

c, Western blot analysis of FRα levels in SUM159PT and HeLa cells treated with PD-L1 FolTAC and TfR1 FolTAC, respectively.

d, Time course of HER2 degradation in SKBR3 treated with HER2 FolTAC v0.2.

e, A schematic illustration of the signaling pathway initiated by the dimerization of HER2 receptors. Upon dimerization, the HER2 receptor is activated, as indicated by the phosphorylation (P) of tyrosine residues in its cytoplasmic domain. This activation leads to the recruitment and activation of phosphoinositide 3-kinase (PI3K). Activated PI3K then facilitates the activation of AKT, a crucial signaling molecule in cell survival and growth pathways, by phosphorylation at two key residues: serine 473 (Ser473) and threonine 308 (Thr308).

f, Western blot analysis of AKT phosphorylation, p-AKT (T308) and p-AKT (S473), upon the addition of affibody control protein.

g, Western blot analysis of AKT phosphorylation, p-AKT (T308) and p-AKT (S473), upon the addition of HER2 FolTAC. SKBR3 cells were treated with either control affibody or HER2 FolTAC for 36 hours.

Created in BioRender. WANG, Z. (2025) https://BioRender.com/d5n9vj6.

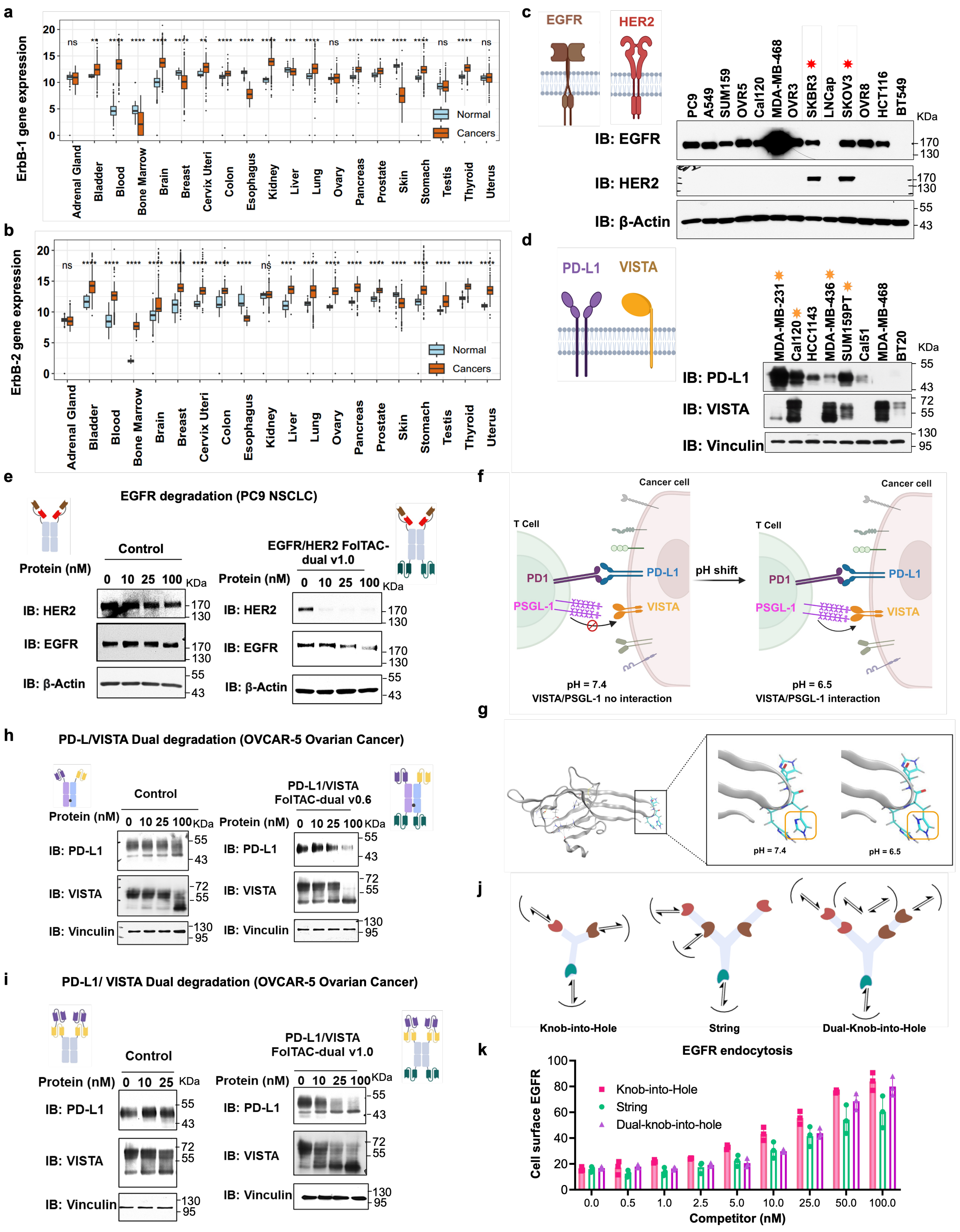

Supplementary Fig. 4. Evaluation of EGFR, HER2, PD-L1, and VISTA expression levels across different cancer tissues and cell lines, as well as the development of FolTAC-dual for dual membrane protein degradation.

a-b, Transcriptomic profile of (a) ErbB-1 and (b) ErbB-2 expression in different tumor and normal tissues. Detailed abbreviations, full names of each cancer subtype, and the standard deviation of the calculations are included in a supplementary Excel file. Exact P value for Supplementary Fig. 4a and 4b are provide in Zenodo under the DOI: https://doi.org/10.5281/zenodo.16421330. Normal cells are indicated in blue, and cancer cells are indicated in orange.

c, Western analysis of EGFR and HER2 co-expression across various cell lines. The red star indicates the cell line that expresses both EGFR and HER2 proteins.

d, Western analysis of PD-L1 and VISTA co-expression across various cell lines. The yellow star indicates the cell line that expresses both PD-L1 and VISTA proteins.

e, Western blot analysis of EGFR and HER2 degradation in PC9 cells mediated by EGFR and HER2 FolTAC-dual v1.0. Left: Cell samples treated with a bispecific control antibody; Right: Cell samples treated with bispecific EGFR/HER2 FolTAC-dual.

f-g, The structure analysis of VISTA at various pH levels. (f) At a physiological pH of 7.4, PSGL-1 does not interact with VISTA. A shift to the acidic tumor microenvironment at pH 6.5 induces an interaction between PSGL-1 and VISTA. This highlights the role of acidic pH in modulating immune checkpoint interactions, potentially affecting T-cell function and cancer immune evasion. (g) The conformational changes in a histidine residue due to pH variation are illustrated. The structural model on the left shows the histidine side chain at a neutral pH of 7.4, where it remains unprotonated. The same histidine residue is depicted at a pH of 6.5, indicative of the acidic tumor microenvironment, where it becomes protonated. This protonation can lead to altered molecular interactions, highlighting the influence of pH on protein behavior and function.

h, Western blot analysis of PD-L1 and VISTA degradation in OVCAR-5 cells mediated by the PD-L1/VISTA FolTAC-dual v0.6.

i, Western blot analysis of PD-L1 and VISTA degradation in OVCAR-5 cells mediated by PD-L1/VISTA FolTAC-dual v1.0.

j, The scheme of protein design for Knob-into-Hole, String, Dual-Knob-into-Hole.

k, EGFR receptor detected via flow cytometry (Detection antibody: V5-647) upon treating with increasing concentrations of EGFR affibody competitor. Cell surface EGFR levels were measured following treatment with increasing concentrations (0-100 nM) of different bispecific formats: knob-into-hole (magenta squares), string (green circles), and dual-knob-into-hole (purple triangles). Data are presented as mean ± SD from n = 3 independent biological replicates. Each data point is overlaid on bar charts.

Created in BioRender. WANG, Z. (2025) https://BioRender.com/d5n9vj6.

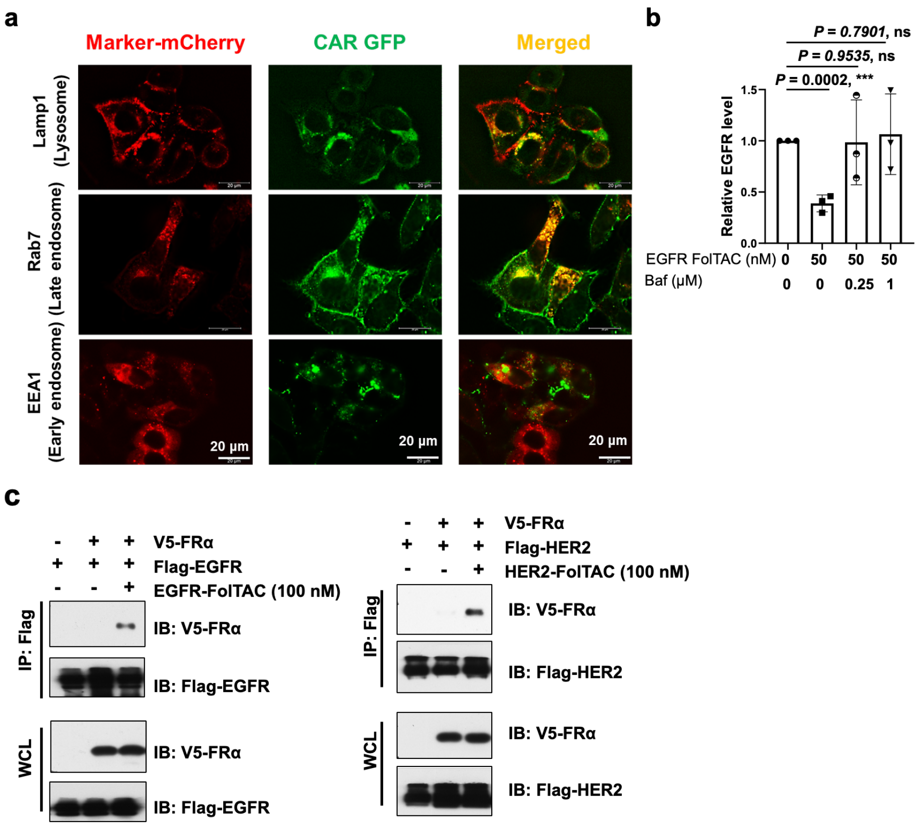

Supplementary Fig. 5. Molecular mechanism underlying FolTAC-mediated protein degradation.

a, Co-localization imaging of CAR-GFP and various endosome markers. Scale bar: 20 μm (white).

b, Quantification of EGFR degradation level by EGFR FolTAC in PC9 cells using the lysosome inhibitor bafilomycin (Baf). Data are shown as mean ± SD. n = 3 independent biological replicates. Statistical significance was determined using two-sided unpaired Student's t-tests. Exact *p*-values are indicated above the bars. $p < 0.05$ was considered significant. ns, not significant; ***, $p < 0.001$.

c, Co-IP experiments to demonstrate ternary complex formation between EGFR/HER2 and FRα mediated by EGFR/HER2 FolTAC.

a

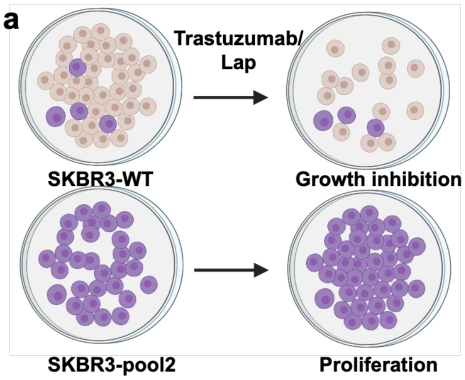

b

SKBR3-WT	IC_{50} (nM)	R^2
Trastuzumab	37.13	0.8981
Lapatinib	7.996	0.8956
Combo	0.844	0.9472

Traz/Lap-resistant SKBR3	IC_{50} (nM)	R^2
Trastuzumab	> 500	-
Lapatinib	87.82	0.9272
Combo	0.734	0.9341

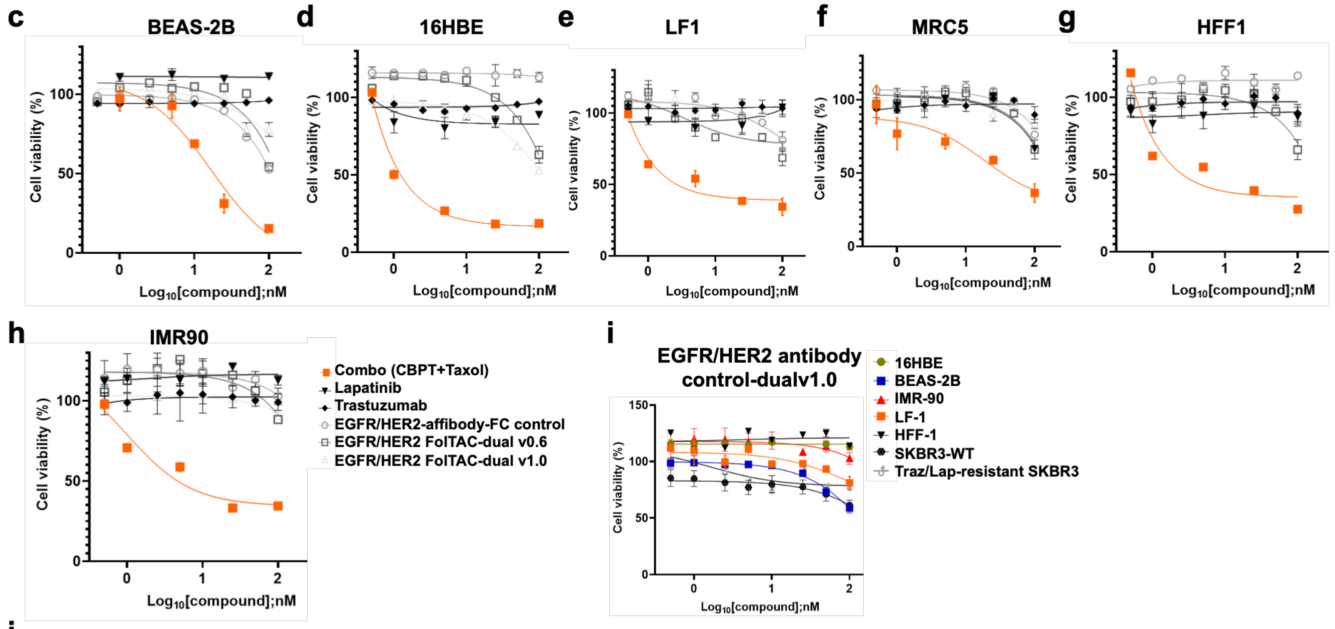

j

	Combo		EGFR/HER2 FolTAC-dual v1.0		EGFR/HER2 antibody control-dual v1.0	
Cell line	IC_{50} (nM)	R^2	IC_{50} (nM)	R^2	IC_{50} (nM)	R^2
16-HBE	1.245	0.9507	> 100	–	> 100	–
BESA-2B	16.74	0.9362	> 100	–	> 100	–
IMR90	6.9814	0.9274	> 100	–	> 100	–
LF-1	4.308	0.7599	> 100	–	> 100	–
HFF-1	3.265	0.9205	> 100	–	> 100	–
SKBR3-WT	0.844	0.9472	12.51	0.9762	> 100	–
Traz/Lap-resistant SKBR3	0.734	0.9341	25.85	0.9795	> 100	–

Supplementary Fig. 6. The cell viability dose curves for various cell lines treated with the indicated compounds and degraders.

a, Schematic illustration of the development of Traz/Lap-resistant SKBR3 cells from parental SKBR3-WT cells through prolonged exposure to trastuzumab and lapatinib.

b, Table of IC_{50} values for Trastuzumab, Lapatinib, and the combination on SKBR3-WT and Trastuzumab/Lapatinib-resistant SKBR3 cells.

c-h, Dose-response curves of (c) BEAS-2B, (d) 16HBE, (e) LF1, (f) MRC-5, (g) HFF-1, (h) IMR-90 that were treated with different drugs for 6 days. The analysis was performed using the Cell Counting Kit-8 (CCK-8) protocol. Data are shown as mean ± SD. n = 3 independent biological replicates. Dose–response curve of various cell lines is treated with different compounds, including combination chemotherapy (CBPT + Taxol, orange squares), Lapatinib (black

triangles), Trastuzumab (black squares), EGFR/HER2-affibody-FC control (open gray circles), EGFR/HER2 FolTAC-dual v0.6 (open grey squares), and EGFR/HER2 FolTAC-dual v1.0 (open gray triangles). CBPT: Carboplatin.

i, Cell viability curves of seven different cell lines (BEAS-2B (blue circles), 16HBE (yellow diamonds), LF1 (red triangles), HFF-1 (black inverted triangles), IMR-90 (orange squares), SKBR3-WT (black circles), and Traz/Lap-resistant SKBR3 (grey crosses)) treated with the EGFR/HER2 bispecific affibody control dual v1.0. Data are shown as mean ± SD. n = 3 independent biological replicates.

j, Table summarizing IC_{50} values of each drug (chemotherapy combo, EGFR/HER2 FolTAC-dual v1.0, and EGFR/HER2 antibody control) on BEAS-2B, 16HBE, LF1, MRC-5, HFF-1, IMR-90, SKBR3, and Traz/Lap-resistant SKBR3 cell lines.

Created in BioRender. WANG, Z. (2025) https://BioRender.com/d5n9vj6.

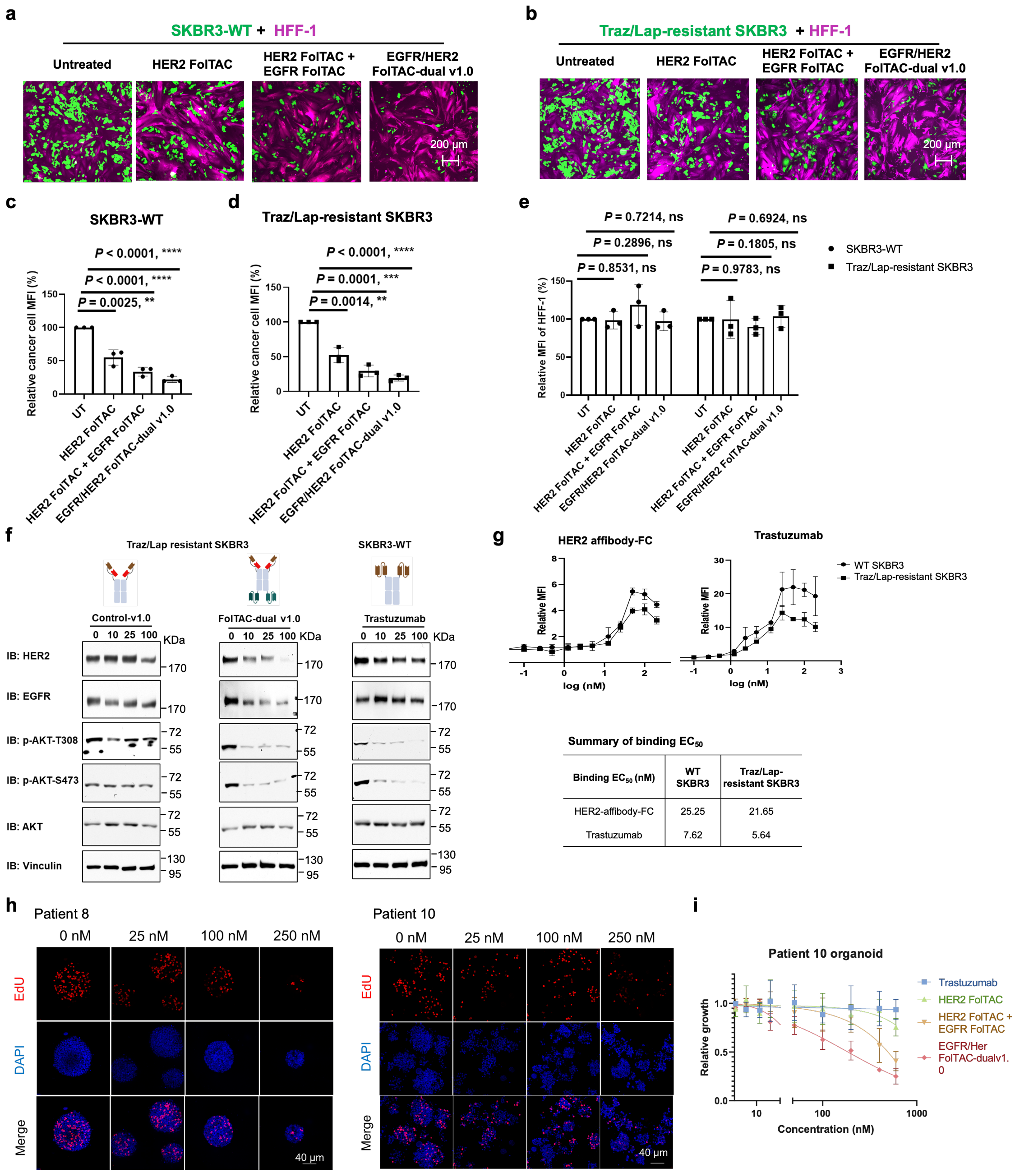

Supplementary Fig. 7. Evaluation of FolTAC-dual as a novel therapeutic strategy to overcome Trastuzumab/Lapatinib resistance in breast cancer using co-culture systems and patient-derived organoid (PDO) models.

a, Evaluation of anti-cancer efficacy and specificity of EGFR/HER2 FolTAC-dual v1.0 in a co-culture assay of SKBR3-WT cancer cells and HFF-1 healthy cells, in comparison to HER2 FolTAC, as well as EGFR FolTAC and HER2 FolTAC combinations. SKBR3-WT cells and HFF-1 cells express GFP (green) and mCherry (magenta),

respectively. Cell morphology and spatial distribution were monitored by fluorescence microscopy. Scale bar: 200 μm (white).

b, Experiments following the same protocol in (a) with Traz/Lap-resistant SKBR3 cancer cells (green) and HFF-1 healthy cell (magenta) co-cultures. EGFR/HER2 FolTAC-dual v1.0 specifically inhibit Traz/Lap-resistant SKBR3 cancer cells while sparing HFF-1 cells. Cells were treated with the drugs at 100 nM for 6 days. Scale bar: 200 μm (white).

c, Quantification analysis of the Mean Fluorescent Intensity (MFI) between SKBR3-WT-GFP and HFF-1-mCherry in Supplementary Fig. 7a.

d, Quantification analysis of the Mean Fluorescent Intensity (MFI) between Traz/Lap-resistant SKBR3-GFP and HFF-1-mCherry in Supplementary Fig. 7b.

e, Quantification analysis of HFF-1-mCherry Mean Fluorescent Intensity (MFI) in Supplementary Fig. 7a and Fig. 7b. Each data point is presented from triplicate independent experiments. The MFIs were quantified using ImageJ software. Data are shown as mean ± SD in Supplementary Fig 7c, 7d, and 7e. n = 3 independent biological replicates. Statistical significance was determined using two-sided unpaired Student's t-tests. Exact *p*-values are indicated above the bars. Differences were considered statistically significant at $p < 0.05$. ns, **, ***, ****: no significance, $p < 0.01$, $p < 0.001$, $p < 0.0001$.

f, Western blot analysis of AKT phosphorylation, p-AKT (T308) and p-AKT (S473), in Traz/Lap-resistant SKBR3 upon adding bispecific affibody-FC control to Traz/Lap-resistant SKBR3 (left panel), EGFR/HER2 FolTAC-dual v1.0 to Traz/Lap-resistant SKBR3 (middle panel), or Trastuzumab to SKBR3-WT (right panel).

g, Plot of Mean Fluorescence Intensity (MFI) of the SKBR3-WT and Traz/Lap-resistant SKBR3 cells treated with HER2-affibody-FC and Trastuzumab at different concentrations. The signal was detected by a secondary antibody conjugated with protein A-647. Data are presented from duplicate experiments. A summary of the binding affinity is represented by EC_{50} values for the affibody-FC and Trastuzumab to both SKBR3-WT and Traz/Lap-resistant SKBR3. Data are shown as mean ± SD. n = 2 independent biological replicates.

h, Fluorescent Images of EdU (Red), DAPI (Blue)-stained PDOs from patients 8 and 10 after a single 7-day treatment with EGFR/HER2 FolTAC-dual v1.0, without refreshing the drug. Magenta marks EdU-positive nuclei. Scale bar: 40 μm (white).

i, Dose-response curve of PDOs from Patient 10 under different treatment groups: Trastuzumab, HER2 FolTAC, HER2 FolTAC combined with EGFR FolTAC, and EGFR/ER2 FolTAC-dual v1.0. Data are shown as mean ± SD. n = 3 independent biological replicates.

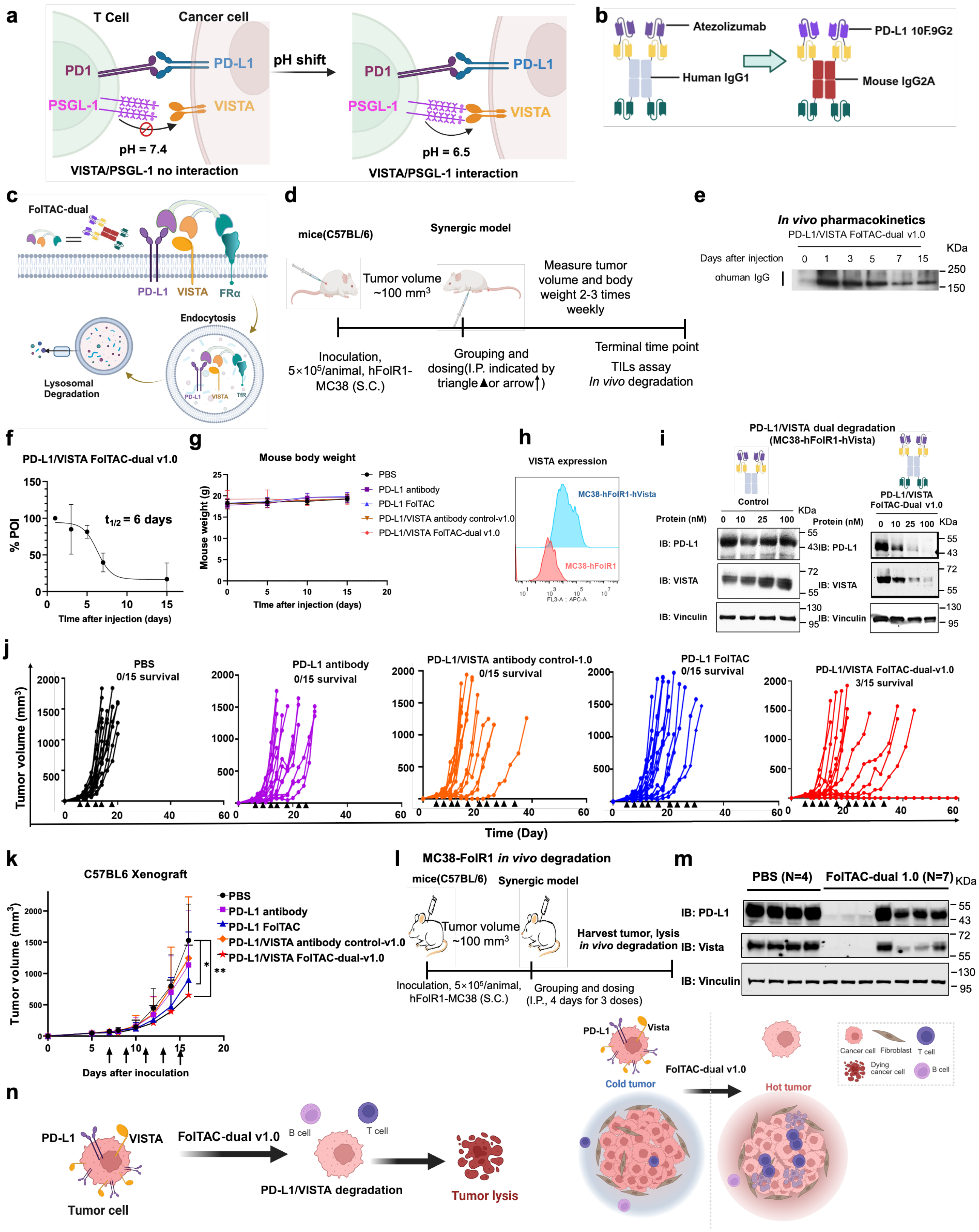

Supplementary Fig. 8. ***In vivo*** **characterization of PD-L1/ VISTA FolTAC-dual v1.0 and its anti-cancer effects through immune activation.**

a, Schematic illustration of the differential interactions between PSGL-1 on T cells and VISTA on cancer cells at varying pH levels. On the left, at a physiological pH of 7.4, PSGL-1 does not interact with VISTA. On the right, a shift to an acidic tumor microenvironment at pH 6.5 induces an interaction between PSGL-1 and VISTA.

b. Re-engineering PD-L1/VISTA FolTAC-dual v1.0 involves creating a chimeric mouse IgG2A variant for studies using syngeneic mouse models. Atezolizumab and a human IgG1 are substituted with PD-L1 10F9.G2 and mouse IgG2A, respectively.

c, Scheme of the process of PD-L1/VISTA FolTAC-dual v1.0 mediated dual degradation of PD-L1 and VISTA.

d, Scheme of the process of tumor inoculation in C57BL/6 mice, followed by the progression of tumor growth and subsequent drug treatments. Tumor Inoculation: A C57BL/6 mouse is inoculated with MC38-*FOLR1*-h*VISTA* tumor cells via subcutaneous injection. Tumor Growth Monitoring: After inoculation, the tumor is allowed to grow until it reaches 100 mm^2 before being subjected to drug treatments.

e-f, Western blot (e), and triplicate quantification (f) of plasma levels of the injected proteins in mice over different days of blood collection are described in (e). Results show that the plasma half-life of PD-L1/VISTA FolTAC-dual v1.0 is comparable to that of EGFR/HER2 FolTAC-dual v1.0. The error bars represent the standard deviation. Data are shown as mean ± SD. n = 3 independent biological replicates.

g, Weight monitoring of mice over time following treatments. n = 3 per treatment group. Mice were treated with PBS, PD-L1 antibody, PD-L1/VISTA antibody control v1.0, PD-L1 FolTAC, and PD-L1/VISTA FolTAC-dual v1.0, respectively. Data are shown as mean ± SD. n = 3 independent biological replicates.

h, Flow cytometry analysis of human VISTA expression in MC38-hFOLR1 engineered cells that express VISTA on the cell surface.

i, Western blot analysis of PD-L1 and VISTA *in vitro* degradation in MC38-h*FOLR1*-hVISTA cells mediated by PD-L1/VISTA FolTAC-dual v1.0. Left: Cell samples treated with bispecific control antibody or affibody-FC; Right: Cell samples treated with bispecific PD-L1/VISTA FolTAC-Dual-v1.0.

j, Tumor growth curve of C57BL6 mice bearing tumor derived from MC38-h*FOLR1*-hVISTA cells upon administration with PBS, PD-L1 antibody, PD-L1/VISTA bispecific antibody control, PD-L1 FolTAC, and PD-L1/VISTA FolTAC-dual v1.0. Black triangles on the x-axis indicate the time points of drug injection. Tumor volume was measured every two days following treatment initiation. n = 15 mice.

k, Tumor growth curve for each drug treatment group shows the changes in tumor size from individual mice. Treatment groups include: PBS (black circles); PD-L1 antibody (purple squares); PD-L1 FolTAC (blue triangles); PD-L1/VISTA antibody control v1.0 (orange inverted squares); PD-L1/VISTA FolTAC-dual v1.0 (red stars). Data are shown as mean ± SD. n = 15 independent biological replicates. Statistical significance was determined using two-sided unpaired Student's t-tests. Differences were considered statistically significant at $p < 0.05$. *, **: $p < 0.05$ ($P = 0.0105$), $p < 0.01$ ($P = 0.0020$).

l, Schematic summary of anti-tumor mechanisms mediated by VISTA/PD-L1 FolTAC-dual v1.0.

m, Western blot analysis of PD-L1 and VISTA degradation *in vivo* in MC38-h*FOLR1*-h*VISTA* cells mediated by PD-L1/VISTA FolTAC-dual v1.0.

n, A schematic illustration of the cytotoxic lysis of tumors mediated by FolTAC-dual. Additionally, it illustrates the transformation of a tumor microenvironment from “cold” to “hot” using the therapeutic FolTAC-dual v1.0, which degrades PD-L1 and VISTA on tumor cells. The progression from a cold tumor to a hot tumor ultimately results in tumor lysis, demonstrating the potential therapeutic impact of modulating the tumor microenvironment.

Created in BioRender. WANG, Z. (2025) https://BioRender.com/d5n9vj6.

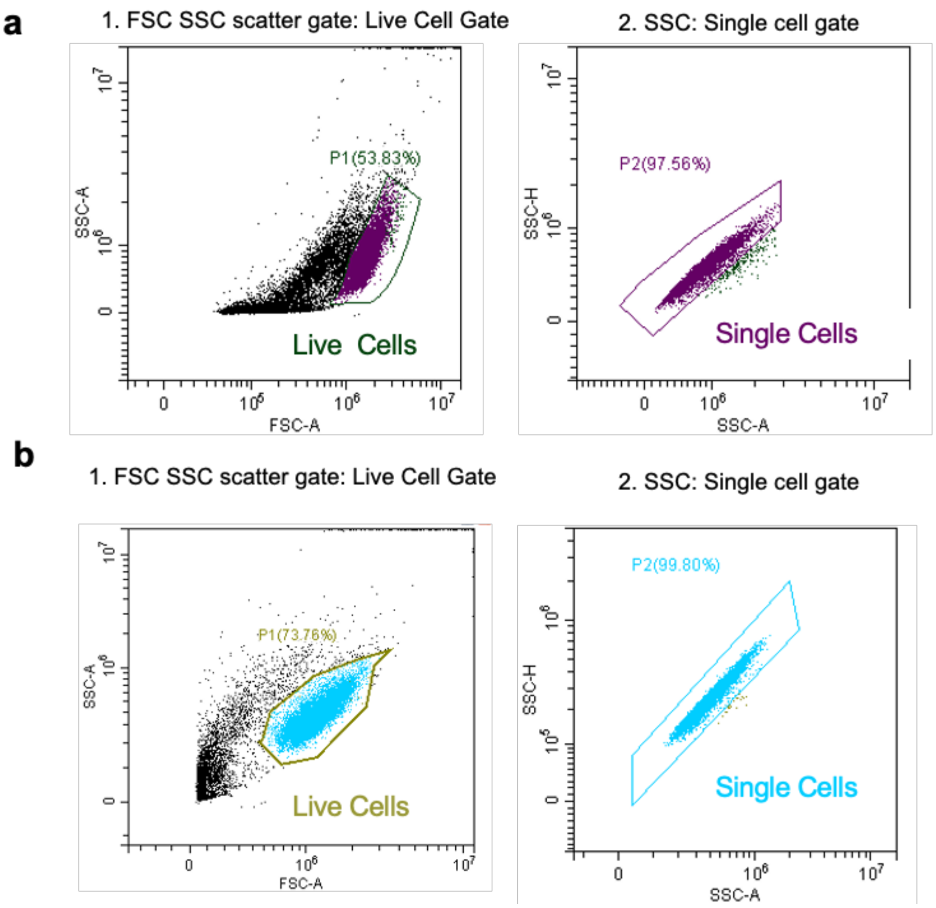

Supplementary Fig. 9. Gating strategies used for FolR1 expression level analysis in different cell lines and primary cells.

a, Gating strategies used for FolR1 expression level analysis in adhesion cells using HeLa as an example. This FACS accounts for all adhesion cell sequential gating strategies including Fig. 1c, Fig. 2j, Fig. 3b, Fig. 3f, Fig. 3h, Fig. 4f-4h, Supplementary Fig. 7c-e, Supplementary Fig. 7g, and Supplementary Fig. 8h.

b, Gating strategies used for FolR1 expression level analysis in suspension cells using primary T cells as an example. This FACS accounts for all suspension cell sequential gating strategies including Fig. 1c.
