# Supplementary material for: Dual membrane receptor degradation via folate receptor targeting chimera
Source: Nat Commun. 2025 Oct 2;16:8804. doi: 10.1038/s41467-025-63882-5 (PMC12491540; doi:10.1038/s41467-025-63882-5)
Supplement: Supplementary file 2 — Reporting Summary [file 41467_2025_63882_MOESM2_ESM.pdf]

nature portfolio

Corresponding author(s): Wenyi Wei
Dingpeng Zhang

Last updated by author(s): Jul 28, 2025

Reporting Summary

Nature Portfolio wishes to improve the reproducibility of the work that we publish. This form provides structure for consistency and transparency in reporting. For further information on Nature Portfolio policies, see our Editorial Policies and the Editorial Policy Checklist.

Statistics

For all statistical analyses, confirm that the following items are present in the figure legend, table legend, main text, or Methods section.

n/a	Confirmed	
☐	☒	The exact sample size (*n*) for each experimental group/condition, given as a discrete number and unit of measurement
☐	☒	A statement on whether measurements were taken from distinct samples or whether the same sample was measured repeatedly
☐	☒	The statistical test(s) used AND whether they are one- or two-sided<br>*Only common tests should be described solely by name; describe more complex techniques in the Methods section.*
☒	☐	A description of all covariates tested
☒	☐	A description of any assumptions or corrections, such as tests of normality and adjustment for multiple comparisons
☐	☒	A full description of the statistical parameters including central tendency (e.g. means) or other basic estimates (e.g. regression coefficient) AND variation (e.g. standard deviation) or associated estimates of uncertainty (e.g. confidence intervals)
☐	☒	For null hypothesis testing, the test statistic (e.g. *F*, *t*, *r*) with confidence intervals, effect sizes, degrees of freedom and *P* value noted<br>*Give P values as exact values whenever suitable.*
☒	☐	For Bayesian analysis, information on the choice of priors and Markov chain Monte Carlo settings
☒	☐	For hierarchical and complex designs, identification of the appropriate level for tests and full reporting of outcomes
☒	☐	Estimates of effect sizes (e.g. Cohen's *d*, Pearson's *r*), indicating how they were calculated

Our web collection on statistics for biologists contains articles on many of the points above.

Software and code

Policy information about availability of computer code

Data collection	CytExpert software version 2.6 was used for data acquisition. Image Studio software (v5.2.5) was used for western blot imaging. Nikon Ti inverted, Zeiss LSM880, or KEYENCE BZ-X700 All-in-One were used for fluorescence microscopy image acquisition.
Data analysis	FlowJo (v 10.8.1) was used for flow cytometry data analysis. ImageJ was used for western blot quantification. ImageJ and Fiji were used for fluorescence microscopy image analysis. GraphPad Prism (v 8.0.2) and Microsoft Excel were used for assay data analysis and graphing. R (version 4.2) was used for transcriptomics data analysis.

For manuscripts utilizing custom algorithms or software that are central to the research but not yet described in published literature, software must be made available to editors and reviewers. We strongly encourage code deposition in a community repository (e.g. GitHub). See the Nature Portfolio guidelines for submitting code & software for further information.

Data

Policy information about availability of data

All manuscripts must include a data availability statement. This statement should provide the following information, where applicable:

- Accession codes, unique identifiers, or web links for publicly available datasets
- A description of any restrictions on data availability
- For clinical datasets or third party data, please ensure that the statement adheres to our policy

All data supporting the findings of this study are available within the article and its Supplementary Information files. Transcriptome data from cancer patients and normal tissues were downloaded from UCSC Xena portal (https://xena.ucsc.edu/). Bioinformatic analysis code for main and supplementary figures have been

deposited in Zenodo under the DOI https://doi.org/10.5281/zenodo.16421330 and is openly accessible under the MIT License. The notebook includes methods for accessing the datasets necessary to interpret, verify, and extend the study, along with clear instructions for reuse and reproducibility. No additional restrictions apply.
Source data are provided with this paper.

Research involving human participants, their data, or biological material

Policy information about studies with human participants or human data. See also policy information about sex, gender (identity/presentation), and sexual orientation and race, ethnicity and racism.

Reporting on sex and gender	Only female-derived breast cancer PDOs were used. Sex was considered during study design to align with the HER2+ breast cancer. All samples were from female patients. Sex and/or gender of participants was determined based on self-report. No sex-disaggregated analyses were performed due to single-sex design.
Reporting on race, ethnicity, or other socially relevant groupings	Race or ethnicity data were not collected for the organoid donors. No analyses based on these characteristics were performed.
Population characteristics	All PDOs were derived from adult female patients with HER2+ breast cancer. Specific age ranges were not disclosed. All samples were obtained under IRB-approved protocols with informed consent.
Recruitment	Tumor tissues were collected from consenting patients at the time of clinical treatment under Dana-Farber/Harvard Cancer Center IRB protocol #17-627. No compensation was provided.
Ethics oversight	Dana-Farber/Harvard Cancer Center Institutional Review Board (IRB protocol #17-627).

Note that full information on the approval of the study protocol must also be provided in the manuscript.

Field-specific reporting

Please select the one below that is the best fit for your research. If you are not sure, read the appropriate sections before making your selection.

- [x] Life sciences
- [ ] Behavioural & social sciences
- [ ] Ecological, evolutionary & environmental sciences

For a reference copy of the document with all sections, see nature.com/documents/nr-reporting-summary-flat.pdf

Life sciences study design

All studies must disclose on these points even when the disclosure is negative.

Sample size	Statistical methods were not used to predetermine sample size. Sample size followed common standard of n=3 or more biological replicates. For mouse experiments, the number of animals used for each experiment was estimated based on the variations of the assays. In our experience, there is low variability for the parameters measured (weight change and serum half-life). A minimum of n=3 replicates is therefore used for these experiments.<br>For the immunology-related animal experiments, 15 female mice were used per group. The sample size was determined based on prior studies conducted in our laboratory (PMID: 29160310; PMID: 32839551, PMID: 33909988), which demonstrated that this number provides sufficient statistical power to detect biologically meaningful differences in tumor response and immune activation.<br>For the xenograft animal experiments for EGFR/HER2 degrader-dual, 7 female mice for each group were selected based on the published work using SKBR3 breast cancer xenograft model (PMID: 37092695; PMID:37370010).
Data exclusions	No data was excluded from this study.
Replication	All experiments were replicated with at least two biological replicates.
Randomization	The choice of mice for injection of different concentrations of FolTACs or controls was random. No other experiments were randomized. Randomization was not relevant to other experiments as this is an observational study which would not be affected by the relevant bias. To counter for potential batch effect, all in vitro treatment were performed on the same plate.
Blinding	Blinding during experimental procedures were not required because experimental conditions could be easily identified from the data itself. Data was first processed in a blinded mode, followed by sample/control assignments.

Reporting for specific materials, systems and methods

We require information from authors about some types of materials, experimental systems and methods used in many studies. Here, indicate whether each material, system or method listed is relevant to your study. If you are not sure if a list item applies to your research, read the appropriate section before selecting a response.

Materials & experimental systems

n/a	Involved in the study
	☒ Antibodies
	☒ Eukaryotic cell lines
☒	Palaeontology and archaeology
	☒ Animals and other organisms
☒	Clinical data
☒	Dual use research of concern
☒	Plants

Methods

n/a	Involved in the study
☒	ChIP-seq
	☒ Flow cytometry
☒	MRI-based neuroimaging

Antibodies

Antibodies used

Anti-HER2 Rabbit pAb (29D8), Cell Signaling Technology; 2165; Dilution 1: 1000; Anti-EGFR Rabbit pAb, ABclonal Science; A21385; Dilution 1: 1000 ; Anti-CD71 Rabbit mAb (D7G9X), Cell Signaling Technology; 13113; Dilution 1: 1000; Anti-PD-L1 Rabbit mAb(E1L3N) Cell Signaling Technology; 13684T; Dilution 1: 1000; Anti-VISTA Rabbit mAb(D5L5T) Cell Signaling Technology; 54979; Dilution 1: 1000; Anti-Folate Receptor Alpha/FOLR1 (E8U2F) Mouse mAb, Cell Signaling Technology, 34265; Dilution 1:1000; Purified anti-V5-tag Antibody, Biolegend, 680601, Dilution 1: 1000.
APC-conjugated anti-human FOLR1 (Bio-Techne, Clone #548908, Cat. #FAB5646A, 1:200), purified anti-V5-tag antibody (BioLegend, Cat. #680601, 1:2000), and Alexa Fluor® 647 anti-human IgG Fc (BioLegend, Clone M1310G05, Cat. #410713, 1:1000).

Validation

All commercial antibodies were validated by antibody suppliers as detailed on each suppliers' website. All in house antibodies and degraders, such as EGFR FolTAC, HER2 FolTAC, VISTA FolTAC, PD-L1 FolTAC, TfR1 FolTAC, HER2 affibody-FC, EGFR/HER2 affibody-FC, EGFR/HER2 FolTAC-dual v1.0, and PD-L1/VISTA FolTAC-dual v1.0, were validated by SDS-page gel analysis and cellular assays.
• Anti-EGFR Rabbit pAb, (ABclonal Science; A21385; 1: 1000): https://abclonal.com/catalog-antibodies/KOValidatedEGFRRabbitpAb/A21385
• Anti-PD-L1 Rabbit mAb(E1L3N) (Cell Signaling; 13684T;1: 1000): https://www.cellsignal.com/products/primary-antibodies/pd-l1-e1l3n-xp-rabbit-mab/13684
• Anti-CD71 Rabbit mAb (D7G9X): (Cell Signaling Technology; 13113S;1: 1000): https://www.cellsignal.com/products/primary-antibodies/cd71-d7g9x-xp-rabbit-mab/13113
• Anti-Her2 Rabbit pAb (29D8) (Cell Signaling Technology; 2165;1: 1000) : https://www.cellsignal.com/products/primary-antibodies/her2-erbb2-29d8-rabbit-mab/2165
• Anti-VISTA Rabbit mAb(D5L5T) (Cell Signaling Technology; 54979;1: 1000): https://www.cellsignal.com/products/primary-antibodies/vista-d5l5t-xp-rabbit-mab/54979
•Folate Receptor Alpha/FOLR1 (E8U2F) Mouse mAb (Cell Signaling Technology; 34265;1: 1000): https://www.cellsignal.com/products/primary-antibodies/folate-receptor-alpha-folr1-e8u2f-mouse-mab/34265?srsltid=AfmBOoqSDUHNoEHWtK_UGYd7ILz5ZoFjK4TX1B-jU43mKc62DiucRYnu
•Purified anti-V5-tag Antibody (Biolegend, 680601; 1: 1000):https://www.biolegend.com/en-gb/products/purified-anti-v5-tag-antibody-12318
All antibodies used in this study were obtained from reputable commercial vendors.
Biolegend: "Antibody clones are then tested in a variety of assays to see which applications they are suited for....Thus, the clone cross-validates itself by demonstrating functionality across orthogonal testing methods."
Cell Signaling Technology: "This product has met all of the quality control standards defined by Cell Signaling Technology, Inc."
ABclonal Science: "I mmunofluorescence analysis of HeLa cells using EGFR Rabbit pAb (A21385) at dilution of 1:100."

Eukaryotic cell lines

Policy information about cell lines and Sex and Gender in Research

Cell line source(s)

HEK293T, HFF-1, IMR-90, HeLa, SKOV3, A549, SUM159PT, HCT116, HCC1806-1, SKBR3, MDA-MB-468, MDA-MB-231, H1299, PC9, LNCAP, BT474, BT549, CAL-120, OVCAR-3, OVCAR-8, MCF-7, HCC1937, 16HBE, and HCC1143 cell lines are maintained from Wenyi Wei Lab cell bank (Beth Israel Deaconess Medical Center). 16HBE are maintained from Ting Wu. The SKBR3-pool2 cell line is a gift from Bolin Liu lab. Primary T cells (female) and Primary B cells (female) were cultured in RPMI media supplemented with 10% FBS. The SKBR3-GFP stable cells, HFF-1 mCherry stable cells, and the Hela CAR-GFP/mCherry- Lamp1 cells were generated in the Wei Lab using standard lentiviral protocols.

In our study, only female cell lines were used in almost all in vitro experiments. This decision was based on scientific rationale related to the breast cancer. No comparisons between sexes were made, and sex-specific effects were not the focus of the current investigation.

Authentication

Cell lines were authenticated by the supplier using standard genotyping methods.

Mycoplasma contamination

The cell lines were not tested for mycoplasma contamination. /All cell lines are routinely tested for mycoplasma and no mycoplasma contamination was reported for parental cell lines utilized.

Commonly misidentified lines
(See ICLAC register)

No misidentified lines were used in this study.

Animals and other research organisms

Policy information about studies involving animals; ARRIVE guidelines recommended for reporting animal research, and Sex and Gender in Research

Laboratory animals	Female Ncr nude mice (NCRNU-F, 4 weeks old) were purchased from Taconic Biosciences, and female C57BL/6J mice (Strain #:000664, 6 weeks old) were purchased from The Jackson Laboratory. Mice were housed in ventilated cages (up to 5 per cage) under standard housing conditions, including a 12:12-h dark/light cycle, ambient temperature (~20–22°C), and humidity (~50%).
Wild animals	No wild animals were involved in this study.
Reporting on sex	In our study, only female mice were used throughout all in vivo experiments. This decision was based on scientific rationale related to the breast cancer model employed, as well as to ensure consistency and reduce variability across groups. No comparisons between sexes were made, and sex-specific effects were not the focus of the current investigation.
Field-collected samples	No field-collected samples were involved in this study.
Ethics oversight	All animal care and experimentation were conducted in full accordance with BIDMC Institutional Animal Care and Use Committee (IACUC) protocol 019-2021-24.

Note that full information on the approval of the study protocol must also be provided in the manuscript.

Plants

Seed stocks	*Report on the source of all seed stocks or other plant material used. If applicable, state the seed stock centre and catalogue number. If plant specimens were collected from the field, describe the collection location, date and sampling procedures.*
Novel plant genotypes	*Describe the methods by which all novel plant genotypes were produced. This includes those generated by transgenic approaches, gene editing, chemical/radiation-based mutagenesis and hybridization. For transgenic lines, describe the transformation method, the number of independent lines analyzed and the generation upon which experiments were performed. For gene-edited lines, describe the editor used, the endogenous sequence targeted for editing, the targeting guide RNA sequence (if applicable) and how the editor was applied.*
Authentication	*Describe any authentication procedures for each seed stock used or novel genotype generated. Describe any experiments used to assess the effect of a mutation and, where applicable, how potential secondary effects (e.g. second site T-DNA insertions, mosiacism, off-target gene editing) were examined.*

Flow Cytometry

Plots

Confirm that:

- [x] The axis labels state the marker and fluorochrome used (e.g. CD4-FITC).
- [x] The axis scales are clearly visible. Include numbers along axes only for bottom left plot of group (a 'group' is an analysis of identical markers).
- [x] All plots are contour plots with outliers or pseudocolor plots.
- [x] A numerical value for number of cells or percentage (with statistics) is provided.

Methodology

Sample preparation	Cells were harvested by centrifugation at 300g for 5 minutes. Cell pellets were washed with cold PBS+1% BSA and centrifuged again at 300g for 5 minutes. Cells were then incubated with primary antibodies in PBS+1% BSA for 10-20 minutes at 4°C. Following this, cells were washed three times with cold PBS+1% BSA, and secondary antibodies (if applicable) were added and incubated for 20 minutes at 4°C. Cells were then washed three times with cold PBS+1% BSA and resuspended in cold PBS for flow analysis.
Instrument	Two Beckman Coulter CytoFLEX LX
Software	CytExpert software version 2.6 was used for data acquisition, FlowJo (v 10.8.1) was used for data analysis.
Cell population abundance	Cell population abundance was determined by first gating live cells using FSC-A and SSC-A, followed by doublet removal using SSC-H vs. SSC-A.
Gating strategy	Gating was performed as follows: FSC-A/SSC-A manual scatter gate on the cell population followed by SSC-H/SSC-A manual gate on single cells. Detailed gating strategies used for each experiment are described in the supplementary materials.

- [x] Tick this box to confirm that a figure exemplifying the gating strategy is provided in the Supplementary Information.
